# Influence of vascular endothelial growth factor single nucleotide polymorphisms on non-small cell lung cancer tumor angiogenesis

**DOI:** 10.3892/or.2012.2075

**Published:** 2012-10-09

**Authors:** AI MAEDA, MASAO NAKATA, KOICHIRO YASUDA, TAKURO YUKAWA, SHINSUKE SAISHO, RIKI OKITA, YUJI HIRAMI, KATSUHIKO SHIMIZU

**Affiliations:** Department of General Thoracic Surgery, Kawasaki Medical School, Kurashiki, Okayama 701-0192, Japan

**Keywords:** polymorphisms, angiogenesis, vascular endothelial growth factor, delta-like ligand 4

## Abstract

Vascular endothelial growth factor (VEGF) plays an important role in tumor angiogenesis. Several studies have reported that genomic *VEGF* polymorphisms may influence *VEGF* synthesis. To evaluate the role of VEGF single nucleotide polymorphisms (SNPs), we examined the expression of several angiogenesis-related proteins [VEGF, hypoxia-inducible factor-1α (HIF-1α) and delta-like ligand 4 (Dll4)] and the spread of microvessels in resected non-small cell lung cancer (NSCLC). Blood and tumor tissue from 83 patients with NSCLC were examined for *VEGF* −460T/C (rs833061) and *VEGF* +405G/C (rs2010963) SNPs using the SNaPshot method. Immunohistochemical staining was performed to measure protein expression and microvessel density (MVD). *VEGF* −460T/C and +405G/C SNPs showed no association with VEGF or HIF-1α expression and MVD. Patients with *VEGF* −460TT and the TC genotype had significantly higher MVD compared to those with the CC genotypes. Furthermore, patients with the *VEGF* −460TT genotype had significantly higher Dll4 expression compared to those with the TC or CC genotypes, while the *VEGF* +405G/C SNP displayed no association with Dll4 expression and MVD. These findings indicate that the *VEGF* −460T/C SNP may have a functional influence on tumor angiogenesis in NSCLC. We hypothesize that *VEGF* SNPs may influence angiogenesis through Dll4.

## Introduction

Angiogenesis plays an important role in tumor progression and metastasis, and vascular endothelial growth factor (VEGF) is a key component. Several studies have demonstrated that VEGF mRNA and protein overexpression are associated with tumor progression and prognosis in non-small cell lung cancer (NSCLC) (1–3).

Several *VEGF* single nucleotide polymorphisms (SNPs) have been recently described (4). *VEGF* is located on chromosome *6p21.3* and is organized into eight exons and seven introns (5,6). The VEGF −460T/C SNP (rs833061) is located in the promoter region and may influence promoter activity (7). Furthermore, the *VEGF* +405G/C SNP (rs2010963) is located within the 5′-untranslated region and may affect transcription factor binding affinity (7,8). These two SNPs have been investigated in different types of cancers, and the association of various *VEGF* SNPs with risk or prognosis of several cancers has been examined (9–12). Recently, *VEGF* +405 and −460 SNPs have been found to be significantly associated with risk and survival in NSCLC (13–15). However, the influence of *VEGF* SNPs on tumor angiogenesis remains unclear. In this study, we examined whether *VEGF* −460 and +405 SNPs may influence VEGF expression and microvessel density (MVD) in NSCLC.

Tumor angiogenesis is influenced by a number of proteins. Hypoxia occurs early in tumor development and results in stable binding of hypoxia-inducible factor-1α (HIF-1α) to DNA and the activation of other angiogenic genes, such as *VEGF*(16,17). Delta-like ligand 4 (Dll4) is a ligand for Notch proteins that is expressed by endothelial cells (18,19) and may be induced by VEGF and HIF-1α (20). It plays an important role in tumor vessel maturation and remodeling (21,22). Therefore, we studied whether these *VEGF* SNPs were associated with the expression of the angiogenesis-related proteins HIF1α and Dll4.

## Patients and methods

### Study population

Blood and tumor samples were obtained from 83 patients with NCSLC who underwent surgical resection at the Kawasaki Medical School Hospital between October, 2008 and December, 2010. The patients did not receive radio- or chemotherapy before surgery. This study was approved by the Ethics Committee of the Kawasaki Medical School, and informed consent was obtained from all patients for the use of their tissue specimens.

### Analysis of VEGF-A −460T/C and +405G/C polymorphisms

Blood samples were collected from all subjects before surgery. Genomic DNA was isolated from peripheral whole blood using the QIAamp™ DNA Blood Mini kit (Qiagen, Hilden, Germany). Genomic regions containing the *VEGF* −460T/C and +405G/C SNPs were amplified by polymerase chain reaction using the following primers: −460T/C, 5′-CGAGAGTGA GGACGTGTGTG-3′ (forward) and 5′-ATTGGAATCCTG GAGTGACC-3′ (reverse); +405G/C, 5′-GAGAGACGGGGT CAGAGAGA-3′ (forward) and 5′-CCCAAAAGCAGGTCAC TCA-3′ (reverse). The VEGF SNPs were genotyped by a single-base primer extension assay using the SNaPshot™ Multiplex kit (Applied Biosystems, Foster City, CA, USA), according to the manufacturer’s instructions. The following primers were used: −460T/C, 5′-ttttttttCTTCTCCCCGCTCCAAC-3′; +405G/C, 5′-tttttttttttttGTGCGAGCAGCGA AAG-3′.

### DNA sequencing

Polymorphism analysis was performed using the ABI PRISM^®^ 310 Genetic Analyzer, and results were evaluated using GeneMapper^®^ software, ver. 4.1 (all were from Applied Biosystems).

### Immunohistochemical staining

VEGF, HIF-1α, Dll4 and CD31 (to measure MVD) expression was analyzed using resected, paraffin-embedded lung cancer tissue. After microtome sectioning (4-μm thick), tissue slides were processed on an automated immunostainer (NexES; Ventana Medical Systems, Tucson, AZ, USA) or manual methods. Streptavidin-biotin-peroxidase detection was performed with diaminobenzidine as the chromogen. The following primary antibodies were used according to the manufacturer’s instructions: VEGF (rabbit polyclonal; sc-152; 1:300 dilution; Santa Cruz Biotechnology, Inc., Santa Cruz, CA, USA), HIF-1α (mouse monoclonal; ESEE122; 1:1,000 dilution; Novus, Littleton, CO, USA), Dll4 (rabbit polyclonal; ab7280; 1:50 dilution; Abcam, Cambridge, MA, USA), and CD31 (mouse monoclonal; 1:50 dilution; Dako, Carpinteria, CA, USA). The slides were examined by two investigators blinded to the corresponding clinicopathological data. The expression of each protein marker was examined and evaluated according to previously reported protocols (1,23–26).

### VEGF staining and scoring

**Figure 1:**
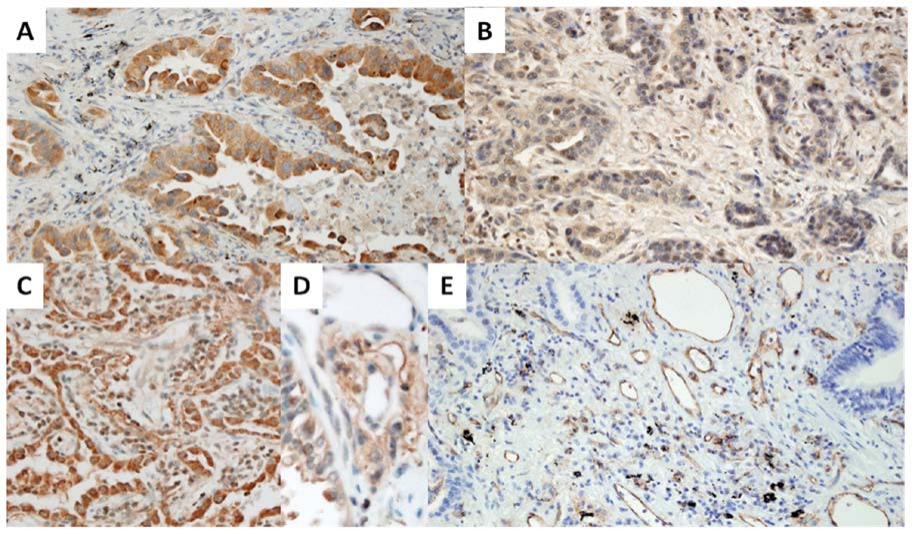
Positive immunohistochemical staining for (A) VEGF, (B) HIF-1α, (C) Dll4 (tumor cells), (D) Dll4 (endothelial cells), and (E) CD31 (for microvessel counting, ×200 magnification).

To evaluate VEGF expression, the percentage of positively stained cells and staining intensity were scored as follows: grade 0, negative; grade 1, weak; grade 2, moderate; grade 3, high; and grade 4, very high (23). Grade 0 indicated staining intensity equal to the negative control, grade 3 indicated intensity equal to the positive control, and grade 4 indicated intensity higher than the positive control. Stain intensity in the cell cytoplasm was similarly scored (23). To determine the percentage of cells with the various staining intensities, the number of immunoreactive cells at each intensity was divided by the total number of tumor cells in three fields at ×200 magnification (Fig. 1A). The overall VEGF staining score was calculated as follows: VEGF score = 1 × percentage of grade 1 cells + 2 × percentage of grade 2 cells + 3 × percentage of grade 3 cells + 4 × percentage of grade 4 cells. The score was analyzed as a continuous and a dichotomous variable.

### HIF-1α staining and scoring

Tumor cells were scored on the intensity and extent of staining as follows: score 1, tumor cells with absent or weak cytoplasmic reactivity and no nuclear reactivity; score 2, tumor cells with moderate/strong cytoplasmic reactivity with a percentage of tumor cells less than their mean percentage and no nuclear reactivity; score 3, tumor cells with moderate/strong cytoplasmic reactivity with a percentage of tumor cells more than their mean percentage; score 4, tumor cells with clear nuclear reactivity (with or without cytoplasmic reactivity regardless of the intensity) (Fig. 1B). Tumors with scores of 1 and 2 were considered to exhibit low HIF-1α expression, whereas those with scores of 2 and 3 were considered to exhibit high HIF-1α expression (24).

### Dll4 staining and scoring

Dll4 expression was considered only in endothelial cells, although recent reports have demonstrated its wide cellular distribution beyond vessels (25,26). To evaluate Dll4 staining in tumor cells (Fig. 1C and D), the intensity of expression was scored on a semiquantitative scale in three ×200 magnification fields. Negative cores were scored as 0, cores with weak expression were scored as 1 and those with moderate/strong expression were scored as 2. High Dll4 expression was defined as a score greater than 1.5 (26).

### Microvessel staining and counting

MVD was assessed by counting the number of microvessels stained for CD31. Vessels with a clearly defined lumen or well-defined linear vessel shape and no single endothelial cells were selected for counting. Microvessels were counted in the three ×200 magnification fields with the highest density (Fig. 1E), and the mean MVD was calculated (1).

### Statistical analysis

Vascular scores were presented as the means ± standard deviation and the difference between the groups was analyzed using the unpaired Student’s t-test. The association of *VEGF* SNPs with clinicopathological parameters and immunostaining results was examined using Chi-squared and Fisher’s exact tests, respectively. The level of significance was set at P<0.05. All analyses were performed using SPSS software (version 17.0; SPSS, Chicago, IL, USA).

## Results

### Clinical characteristics

Characteristics of the patients with NSCLC are summarized in Table I. Patients ranged in age from 49 to 89 years (median, 72 years), with 52 men and 31 women. Fifty-six (67.5%) patients were former/current smokers. There were 40 (48.2%) stage IA, 17 (20.5%) stage IB, 11 (13.3%) stage IIA, 9 (10.8%) stage IIB, 6 (7.2%) stage III. Fifty-two (62.7%) patients had adenocarcinoma, 19 (22.9%) had squamous cell carcinoma, and 12 (14.4%) had other histological malignancies.

### Immunohistochemistry of angiogenesis-related proteins

Forty-two patients (50.6%) exhibited a marked increase in VEGF immunoreactivity of tumor cells. The mean VEGF staining score was 2.79±0.67, and the median score of 2.90 was used to distinguish between low and high VEGF staining. VEGF expression was correlated with HIF1α expression (P=0.003), but not with Dll4 expression (P=0.446) (Table II).

### VEGF SNPs and clinicopathological characteristics

For the *VEGF* +405G/C SNP, 50.6% of patients had the GC genotype, 25.3% had CC and 24.1% had GG. For the *VEGF* −460T/C SNP, 50.6% had the TT genotype, 38.6% had TC and 10.8% had CC. No significant association was observed between *VEGF* SNPs and clinicopathological characteristics such as gender, pathological stage, lymphatic invasion, vascular invasion, histological type, and smoking status (Table III).

### VEGF SNPs and angiogenesis-related proteins

Both SNPs displayed no association with VEGF or HIF-1α expression; however, Dll4 expression was significantly higher in patients with the *VEGF* −460TT genotype (P=0.031) (Table IV).

### Angiogenesis-related proteins and MVD

MVD ranged from 2.0 to 80.0, with a mean value of 29.9±15.9 and a median score of 29. High MVD was significantly associated with high VEGF (P<0.001) and Dll4 (P=0.026) expression, but not with HIF-1α expression (P=0.235) (Table V).

### VEGF SNPs and MVD

Patients with the *VEGF* −460TT and TC genotypes had significantly greater MVD compared to those with the CC genotype (TT/TC vs. CC; P=0.027) (Table VIA). Moreover, in a group of tumors with high VEGF expression, patients with the VEGF −460TT genotype had significantly higher MVD compared to those with the CC genotypes (P=0.033) (Table VIB).

## Discussion

Angiogenesis is important for tumor progression and utilizes several factors, with VEGF being the key factor. Recently, several *VEGF* SNPs have been identified, and their effect has attracted a great deal of attention. An *in vivo* study by Stevens *et al*(7) discovered that *VEGF* −460/+405 SNPs significantly altered VEGF promoter activity in response to phorbol esters. Recent literature has reported the association of *VEGF* SNPs with risk or prognosis of various types of cancers (9–12). A large case-control study in Caucasians demonstrated that male patients with NSCLC and the *VEGF* +405CC+CG genotype had a higher risk of lung adenocarcinoma, while those with the −460T/+405G/936C haplotype had a reduced risk. (14). The C allele of the *VEGF* +405G/C SNP significantly improved survival in early-stage NSCLC (13), whereas the −460CC genotype decreased overall survival in advanced-stage NSCLC (15). Other studies have suggested a lower survival rate for the *VEGF* +405CC genotype in gastric and ovarian cancers (27,28). The reason for these conflicting results is currently unclear, and the influence of *VEGF* SNPs remains uncertain and controversial.

However to date, few studies have focused on the association between *VEGF* SNPs and VEGF expression. Therefore, we conducted a study with NSCLC patients to examine the functional activity of *VEGF* SNPs and their possible role in VEGF expression and angiogenesis.

The genotype frequencies for *VEGF* +405G/C (GG, CC, and GC) and *VEGF* −460T/C (TT, CC and TC) SNPs in this study were equivalent to previous reports involving Japanese patients (4,15). In our current study, there was no association between *VEGF* SNPs and VEGF expression. Koukourakis *et al*(29) reported that *VEGF* SNPs were associated with VEGF expression in NSCLC tumor cells and tumor angiogenic activity. They discovered that the *VEGF* −2578CC, +405GG (also referred to as −634GG) −1154AA and GA genotypes were associated with low VEGF expression in 36 patients with NSCLC (29). The vascular density of patients with the *VEGF* −2578CC and +405GG genotypes was also significantly lower compared to that in patiens with other genotypes. This result is not in agreement with our findings, which may be due to variations in genotype function related to racial differences between the patient groups.

We discovered that patients with the *VEGF* −460TT and TC genotype had significantly higher MVD compared to those with CC genotypes. In general, as in our study, high VEGF expression is associated with high vascular density. However, there was no association between the *VEGF* −460T/C SNP and VEGF expression in tumors. Furthermore, even in high VEGF expression cases, the −460TT genotype was associated with significantly higher MVD compared to CC genotype. This result suggested that high MVD in −460TT genotype was not caused by VEGF expression. The *VEGF* −460TT genotype was associated with significantly higher Dll4 protein expression, which demonstrated a significant association with high MVD. From these results, we concluded that Dll4, induced by the *VEGF* −460TT genotype, influenced the spread of microvessels. Dll4 is generally upregulated by VEGF, which in turn acts as a negative feedback regulator of VEGF. Our results suggest that *VEGF* SNPs may influence VEGF downstream signaling to Dll4, although potential mechanisms have not been examined in this study. Dll4 is associated with tumor vessel maturation and remodeling (21,22). Thus, high Dll4 expression should theoretically lead to fewer but larger vessels, and Dll4 overexpression or inhibition may consequently impair tumor angiogenesis. However, further study of this visceral function is warranted.

In conclusion, the *VEGF* −460T/C SNP may have a functional influence on tumor angiogenesis in NSCLC. Although *VEGF* SNPs were not associated with VEGF expression in tumor cells, they are considered to regulate the response to Dll4 signaling through functional changes in VEGF.

## Figures and Tables

**Table I:** Characteristics of the patients with NSCLC.

Characteristic	No. of patients	%
Age (years)		
Median	72	
Range	49–89	
Gender		
Male	52	62.7
Female	31	37.3
Smoking		
Never	27	32.5
Former/Current	56	67.5
Stage		
IA	40	48.2
IB	17	20.5
IIA	11	13.3
IIB	9	10.8
III	6	7.2
Histology		
Adenocarcinoma	52	62.7
SCC	19	22.9
Other types	12	14.4

SCC, squamous cell carcinoma; NSCLC, non-small cell lung cancer.

**Table II:** Relationships between angiogenesis related protein expression as determined by immunohistochemistry.

	VEGF		HIF-1α	
		
Variable	High	Low	High	Low
HIF-1α				
High	29	15		
Low	13	26		
P-value	P=0.003			
DLL4 (T)				
High	27	23	34	16
Low	15	18	10	23
P-value	P=0.446		P<0.001	

VEGF, vascular endothelial growth factor; Dll4, delta-like ligand 4; HIF-1α, hypoxia-inducible factor-1α; T, tumor cells.

**Table III:** *VEGF* SNPs and clinicopathological characteristics.

	*VEGF* +405 genotype				*VEGF* −460 genotype			
		
Characteristic	CC	GC	GG	P-value	TT	TC	CC	P-value
No. of patients (%)	21 (25.3)	42 (50.6)	20 (24.1)		42 (50.6)	32 (38.6)	9 (10.8)	
Gender								
Male	15	23	14	0.321	23	21	8	0.143
Female	6	19	6		19	11	1	
Stage								
IA	11	19	10	0.807	21	14	5	0.481
IB	5	8	4		7	8	2	
II	3	11	6		9	10	1	
III	2	4	0		5	0	1	
Lymphatic invasion								
+	5	10	3	0.707	10	6	2	0.871
−	16	32	17		32	26	7	
Vascular invasion								
+	10	15	8	0.661	19	10	4	0.455
−	11	27	12		23	22	4	
Histology								
Adenocarcinoma	12	27	13	0.522	26	21	5	0.688
SCC	8	8	3		8	8	3	
Other types	1	7	4		8	3	1	
Smoking								
Never	7	14	6	0.962	17	6	4	0.102
Former/current	14	28	14		25	26	5	

*VEGF*, vascular endothelial growth factor; SCC, squamous cell carcinoma.

**Table IV:** *VEGF* SNPs and angiogenic-related protein expression.

*VEGF* Genotype	VEGF			HIF-1α			Dll4		
		
	High	Low	P-value	High	Low	P-value	High	Low	P-value
*VEGF*+405									
CC	12	9	0.735	10	11	0.739	12	9	0.741
GC	21	21		24	18		27	15	
GG	9	11		10	10		11	9	
*VEGF* −460									
TT	19	23	0.448	21	21	0.289	31	11	0.031
TC	19	13		16	16		14	18	
CC	4	5		7	2		5	4	

Dll4, delta-like ligand 4; HIF-1α, hypoxia-inducible factor-1α; *VEGF*, vascular endothelial growth factor.

**Table V:** Angiogenesis-related protein expression and MVD.

Protein marker expression	MVD	

	Mean ± SD	P-value
VEGF		
High	37.2±18.0	<0.001
Low	24.3±11.7	
Dll4 (T)		
High	33.9±17.4	0.026
Low	26.1±13.9	
HIF-1α		
High	32.9±16.5	0.235
Low	28.5±16.3	

VEGF, vascular endothelial growth factor; Dll4, delta-like ligand 4; HIF-1α, hypoxia-inducible factor-1α; MVD, microvessel density; SD, standard deviation; T, tumor cells.

**Table VI:** *VEGF* SNPs and MVD.

A, *VEGF* SNPs and MVD		

*VEGF* Genotype	MVD	

	Mean ± SD	P-value
*VEGF* +405		
CC	27.3±17.0	CC/GC vs. GG 0.426
GC	31.9±16.4	GG/GC vs. CC 0.961
GG	28.8±14.0	
*VEGF* −460		
TT	31.9±18.1	TC/CC vs. TT 0.550
TC	31.4±16.0	TT/TC vs. CC 0.027
CC	23.9±7.8	

B, *VEGF* SNPs and MVD in the high VEGF expression group		

*VEGF* Genotype	MVD	

	Mean ± SD	P-value

*VEGF* +405		
CC	36.75±19.16	CC/GC vs. GG 0.392
GC	39.48±18.14	CC vs. GG 0.615
GG	32.67±17.29	
*VEGF* −460		
TT	40.05±19.77	TT/TC vs. CC 0.032
TC	36.63±17.54	TT vs. CC 0.033
CC	26.75±6.85	

MVD, microvessel density; SD, standard deviation; VEGF, vascular endothelial growth factor.
